# TGF-β1 upregulates secreted protein acidic and rich in cysteine expression in human granulosa-lutein cells: a potential mechanism for the pathogenesis of ovarian hyperstimulation syndrome

**DOI:** 10.1186/s12964-023-01123-2

**Published:** 2023-05-08

**Authors:** Xuan Dang, Lanlan Fang, Qiongqiong Jia, Ze Wu, Yanjie Guo, Boqun Liu, Jung-Chien Cheng, Ying-Pu Sun

**Affiliations:** Center for Reproductive Medicine Henan Key Laboratory of Reproduction and Genetics The First Affiliated Hospital of Zhengzhou University 40, Daxue Road, Zhengzhou, Henan China

**Keywords:** SPARC, TGF-β1, Granulosa cells, OHSS, Ovary

## Abstract

**Background:**

Ovarian hyperstimulation syndrome (OHSS) is a serious complication during in vitro fertilization (IVF) treatment. The upregulation of ovarian transforming growth factor-beta 1 (TGF-β1) is involved in the development of OHSS. The secreted protein acidic and rich in cysteine (SPARC) is a secreted multifunctional matricellular glycoprotein. Although the regulatory effects of TGF-β1 on SPARC expression have been reported, whether TGF-β1 regulates SPARC expression in the human ovary remains unknown. In addition, the role of SPARC in the pathogenesis of OHSS is unclear.

**Methods:**

A steroidogenic human ovarian granulosa-like tumor cell line, KGN, and primary culture of human granulosa-lutein (hGL) cells obtained from patients undergoing IVF treatment were used as experimental models. OHSS was induced in rats, and ovaries were collected. Follicular fluid samples were collected from 39 OHSS and 35 non-OHSS patients during oocyte retrieval. The underlying molecular mechanisms mediating the effect of TGF-β1 on SPARC expression were explored by a series of in vitro experiments.

**Results:**

TGF-β1 upregulated SPARC expression in both KGN and hGL cells. The stimulatory effect of TGF-β1 on SPARC expression was mediated by SMAD3 but not SMAD2. The transcription factors, Snail and Slug, were induced in response to the TGF-β1 treatment. However, only Slug was required for the TGF-β1-induced SPARC expression. Conversely, we found that the knockdown of SPARC decreased Slug expression. Our results also revealed that SPARC was upregulated in the OHSS rat ovaries and in the follicular fluid of OHSS patients. Knockdown of SPARC attenuated the TGF-β1-stimulated expression of vascular endothelial growth factor (VEGF) and aromatase, two markers of OHSS. Moreover, the knockdown of SPARC reduced TGF-β1 signaling by downregulating SMAD4 expression.

**Conclusions:**

By illustrating the potential physiological and pathological roles of TGF-β1 in the regulation of SPARC in hGL cells, our results may serve to improve current strategies used to treat clinical infertility and OHSS.

Video Abstract

**Supplementary Information:**

The online version contains supplementary material available at 10.1186/s12964-023-01123-2.

## Background

The secreted protein acidic and rich in cysteine (SPARC), also known as osteonectin or BM-40, was first identified in fetal calf bone [1, 2]. SPARC is a multifunctional matricellular glycoprotein that can be secreted by several types of cells. Given its ability to modulate cell-extracellular matrix, SPARC is known to function as a counter adhesive and anti-proliferative protein and can influence cell responses to growth factors [3]. SPARC is highly expressed in tissues undergoing events that require changes in cell–matrix and cell–cell contact, particularly tissue repair or remodeling and embryonic development [4]. The expression of ovarian SPARC has been reported in different species, including rats, cows, sheep, and humans [5–10]. In rat ovaries, examining the spatiotemporal expression pattern of SPARC shows that SPARC protein is detected in granulosa, theca, and stromal cells as well as in oocytes and corpus luteum, and the levels of SPARC mRNA peak during early folliculogenesis, ovulation, and luteolysis [6]. Animal studies have shown that SPARC may play an important modulatory role in regulating angiogenesis during luteal development and maturation [5, 7–9]. However, to date, the regulation and function of SPARC in human ovaries remain largely unknown.

Ovulation and the following luteinization are induced by the mid-cycle luteinizing hormone (LH) surge. The process of ovulation and luteinization involves a series of biochemical and morphological changes that includes tissue repair, tissue remodeling, and neovascularization. Ovarian hyperstimulation syndrome (OHSS) is a serious and iatrogenic complication during the in vitro fertilization (IVF) treatment. OHSS is caused by ovarian stimulation with exogenous gonadotropins and subsequently ovulation induction by human chorionic gonadotropin (hCG) [11]. The hallmarks of OHSS are massive ovarian enlargement together with an increase in capillary permeability, which leads to the fluid shift from the intravascular space to the third space compartments resulting in the development of ascites [12]. Although the pathophysiology of OHSS has not been completely elucidated yet, the vascular endothelial growth factor (VEGF) has been considered a key vasoactive factor in inducing OHSS [13]. In addition to VEGF, high serum estradiol (E2) levels before hCG administration are associated with the development of OHSS [14].

Transforming growth factor-beta 1 (TGF-β1) and its receptors are expressed in human granulosa cells [15, 16]. Studies have revealed that TGF-β1 acts as a local factor that regulates various ovarian functions via autocrine or paracrine fashion [17]. Our previous study shows that the concentration of TGF-β1 in human follicular fluid is higher in OHSS patients than in control patients. TGF-β1 upregulates VEGF expression in human granulosa-lutein (hGL) cells, and that contributes to the development of OHSS [18]. In addition, the expression of aromatase, a key enzyme that mediates E2 synthesis, is upregulated in hGL cells by TGF-β1 and involved in the pathogenesis of OHSS [19–21]. SPARC expression is induced by TGF-β1 in different types of cells [22]. However, whether the expression of SPARC is regulated by TGF-β1 in hGL cells is unknown. During IVF treatment, OHSS does not occur if hCG is not administered. Interestingly, the administration of hCG in pregnant mare serum gonadotropin (PMSG)-primed rats stimulates SPARC expression in granulosa cells [9]. These findings suggest that SPARC may contribute to the pathogenesis of OHSS. Therefore, the present study was designed to explore the effect and related underlying molecular mechanisms of TGF-β1 on SPARC expression in hGL cells and to investigate the biological function of SPARC in OHSS.

## Methods

### Antibodies and reagents

The SPARC (#5420), p-SMAD2 (#3108), p-SMAD3 (#9520), SMAD2 (#3103), SMAD3 (#9523), SMAD4 (#38,454), Snail (#3895), Slug (#9585) antibodies were purchased from Cell Signaling Technology. The aromatase antibody was purchased from Bio-Rad Laboratories (#MCA2077). The VEGF antibody was purchased from Thermo Fisher Scientific (#MA5-13182). The α-tubulin (#sc-23948) antibody was purchased from Santa Cruz Biotechnology. The SPARC (#8725) antibody for immunohistochemistry was purchased from Cell Signaling Technology. The recombinant human TGF-β1 was obtained from R&D systems. The SB431542 was obtained from Sigma.

### Cell culture

The human granulosa cell tumor-derived cell line, KGN [23], was kindly provided by Professor Aaron Hsueh in the Department of Obstetrics and Gynecology at Stanford University. The primary human granulosa-lutein (hGL) cells were purified by density centrifugation from follicular aspirates collected from women undergoing oocyte retrieval, as previously described [24]. Cells were cultured in a humidified atmosphere containing 5% CO_2_ and 95% air at 37 °C in Dulbecco’s Modified Eagle Medium/nutrient mixture F-12 Ham medium (DMEM/F-12; Gibco) supplemented with 10% charcoal/dextran-treated FBS (HyClone), 100 U/mL of penicillin and 100 μg/mL of streptomycin sulfate (Boster). Individual primary cultures were composed of cells from one individual patient. Each experiment was repeated at least three times, and each time used cells derived from different patients or different passages.

### Human follicular fluid samples

The study received approval and was carried out in accordance with the approved guidelines from the Zhengzhou University Research Ethics Board. Written informed consent was obtained from all patients before collecting clinical samples. Human follicular fluid samples were obtained from patients during IVF treatment. None of the patients had been prescribed any medications before enrolment. The causes of infertility were tubal obstruction or male infertility. Patients with polycystic ovary syndrome (PCOS), endometriosis, diminished ovarian reserve, chromosome abnormality, or hydrosalpinx were excluded from the study. According to the symptoms, all OHSS patients were diagnosed with moderate or severe OHSS [25]. All patients were treated with gonadotropin-releasing hormone agonist (GnRH-a) pituitary downregulation protocol. Briefly, on the second day of menstruation, the GnRH-a (3.75 mg) was administered subcutaneously to the patients. Approximately 30 days after GnRH-a injection, recombinant follicle-stimulating hormone (FSH) was administered daily at a dosage of 100–300 IU. When at least three follicles had reached 18 mm, final oocyte maturation was conventionally induced by 6500 IU recombinant hCG (Merck, Germany) and 2000 IU urinary hCG (Livzon, Zhuhai, China). Oocyte retrieval was conducted approximately 37 h after hCG administration by the transvaginal ultrasound-guided follicular aspiration. The follicular fluid sample was collected when the oocytes were retrieved. The follicular fluid sample from each patient was collected as a pool from different follicles. Only the follicular fluid aspirate without blood or flushing solution was collected and analyzed. After 10 min of centrifugation at 1200 rpm, the supernatant was stored at -80 °C until further examination. Detailed information about patients’ characteristics and hormone profiles was extracted from the electronic medical records.

### Rat OHSS model

Ethical approval was obtained from the Zhengzhou University Animal Research Ethics Board for conducting the animal studies. Female Wistar rats were purchased from the Charles River Laboratories (Beijing, China). The Guide for the Care and Use of Laboratory Animals published by the US National Institutes of Health was followed for animal handling. The rats were housed in an environmentally controlled room with free access to food and water. The rat OHSS model was generated based on the protocols described by previous studies [19, 26]. Briefly, PMSG (50 IU/d) was administered i.p. for four consecutive days to 4-week-old rats, followed by hCG administration (25 IU, i.p.) on the fifth day. Control rats were injected with a single dose of PMSG (10 IU) followed by hCG (10 IU) 48 h later. All rats were euthanized on day 7. Each group contained five rats. Changes in body and ovarian weight were measured.

### Reverse transcription quantitative real-time PCR (RT-qPCR)

Total RNA was extracted with TRIzol (Invitrogen) according to the manufacturer’s instructions. RNA (1 μg) was reverse-transcribed into first-strand cDNA with the iScript Reverse Transcription Kit (Bio-Rad Laboratories). Each 20 μL qPCR reaction contained 1 × SYBR Green PCR Master Mix (Applied Biosystems), 60 ng of cDNA, and 250 nM of each specific primer. The primers used in the present study are presented in supplemental Table 1. The qPCR was performed on an Applied Biosystems QuantStudio 12 K Flex system equipped with 96-well optical reaction plates. The specificity of each assay was validated by melting curve analysis and agarose gel electrophoresis of the PCR products. All of the RT-qPCR experiments were run in triplicate, and a mean value was used to determine the mRNA levels. RNase-free water and mRNA without reverse transcription were used as negative controls. Relative quantification of the mRNA levels was performed using the comparative Ct method with GAPDH as the reference gene and using the formula 2^–∆∆Ct^.

### Western blot

Cells were lysed in cell lysis buffer (Cell Signaling Technology) supplemented with a protease inhibitor cocktail (Sigma). The protein concentration was measured by the BCA protein assay kit (Thermo Scientific). Samples with an equal amount of protein were separated by SDS–polyacrylamide gel electrophoresis and transferred to PVDF membranes. After 1 h blocking at room temperature with 5% non-fat dry milk in Tris-buffered saline (TBS), the membranes were incubated overnight at 4 °C with primary antibodies diluted in 5% non-fat milk/TBS. Following primary antibody incubation, the membranes were washed with TBS and subsequently incubated with appropriate HRP-conjugated secondary antibodies (Bio-Rad Laboratories). The immunoreactive bands were detected with an enhanced chemiluminescence kit and imaged with a ChemiDoc MP Imager (Bio-Rad Laboratories).

### Small interfering RNA (siRNA) transfection

To knock down endogenous ALK5, SMAD2, SMAD3, SMAD4, Snail, Slug, or SPARC, cells were transfected with 50 nM ON-TARGETplus SMARTpool siRNA targeting a specific gene (Dharmacon) using Lipofectamine RNAiMAX (Invitrogen). The siCONTROL NON-TARGETING pool siRNA (Dharmacon) was used as the transfection control.

### Immunohistochemistry

Paraffin-embedded sections (5 μm) were deparaffinized and rehydrated. Antigen retrieval was conducted by boiling sections in sodium citrate buffer (pH 6.0) for 8 min. Endogenous peroxidase activity was blocked by incubating sections of 3% hydrogen peroxide solution at room temperature for 10 min. After 1 h of blocking with 3% bovine serum albumin in PBS, sections were incubated with specific primary antibodies overnight at 4 °C. Following primary antibody incubation, the sections were incubated with HRP-conjugated secondary antibody. Sections were developed using the Peroxidase/DAB Dako REAL EnVision Detection System (Dako) and counterstained with hematoxylin. Negative control in the absence of a primary antibody was performed in parallel. Three areas were randomly selected from each section under 200 × magnification, and the integrated optical density values were measured by the Image-Pro Plus 6.0 software. The quantification results were normalized with means of controls.

### ELISA assay for SPARC

SPARC levels in rat serum (10 × dilution) were measured using an ELISA Kit (#NBP2-76,783, Novus Biologicals) as per the manufacturer’s instructions. The interassay CV was < 4.68%. The intraassay CV was < 4.67%. The analytical sensitivity of rat SPARC ELISA was 18.75 pg/mL. SPARC levels in human follicular fluid (50 × dilution) were measured using an ELISA Kit (#DSP00, R&D Systems) as per the manufacturer’s instructions. The interassay CV was ≤ 8.5%. The intraassay CV was ≤ 2.5%. The analytical sensitivity of human SPARC ELISA was 0.269 ng/mL.

### Statistical analysis

The results of animal experiments and clinical data are presented as the mean ± SD. The results of cell experiments are presented as the mean ± SEM of at least three independent experiments. All statistical analyses were analyzed by the PRISM software. Multiple comparisons were analyzed by one-way ANOVA, followed by Tukey’s multiple comparison tests. A significant difference was defined as *p* < 0.05. Values that are statistically different from one another (*p* < 0.05) are indicated by different letters. The values with any common letter are not significantly different.

## Results

### TGF-β1 upregulates SPARC expression in hGL cells

**Fig. 1:**
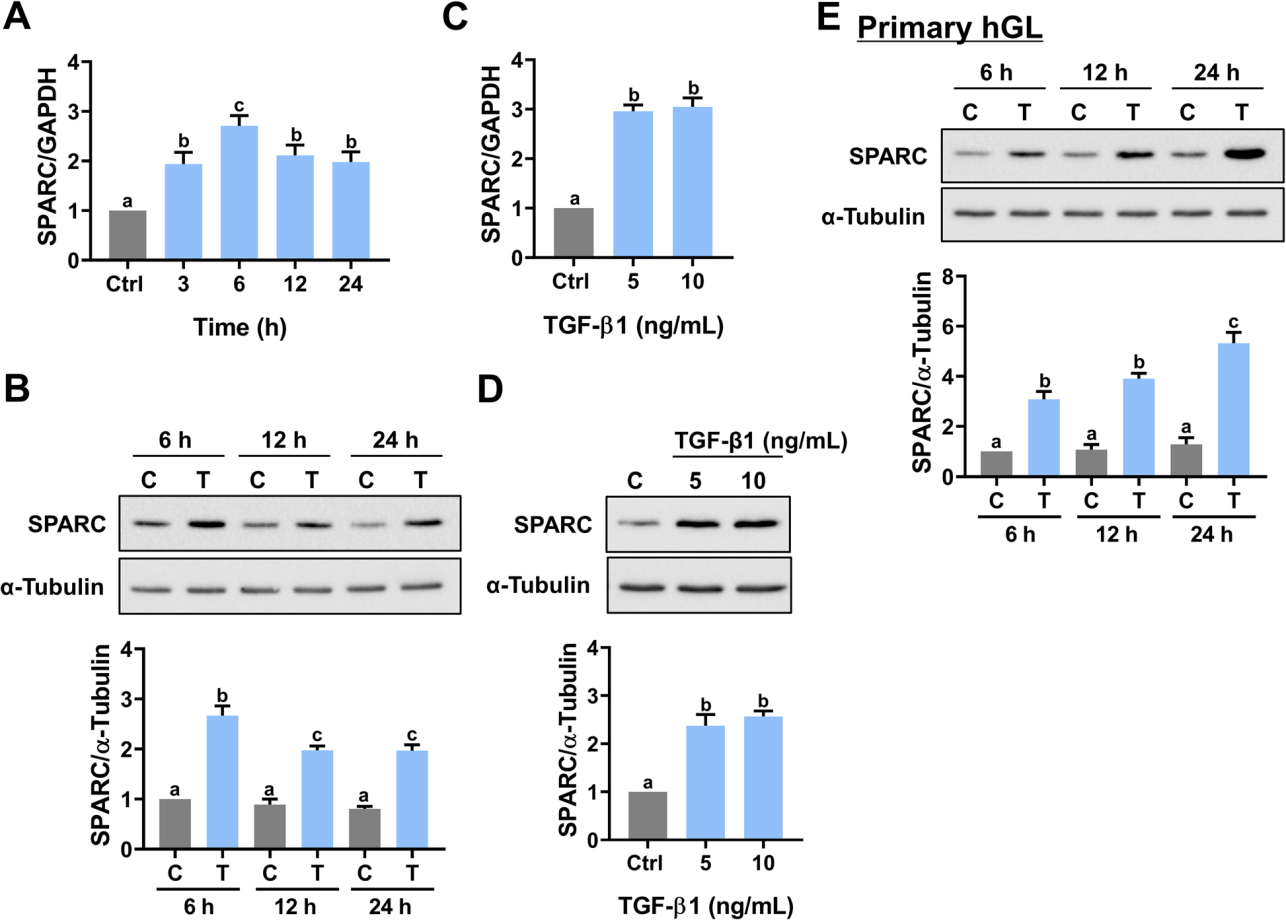
TGF-β1 stimulates SPARC expression in KGN and primary hGL cells. **A** KGN cells were treated with 10 ng/mL TGF-β1 for different periods, and the mRNA levels of SPARC were examined by RT-qPCR. The level of SPARC mRNA at each time point was normalized to the GAPDH mRNA level at the same time point. **B** KGN cells were treated with 10 ng/mL TGF-β1 (T) for 6, 12, and 24 h, and the protein levels of SPARC were examined by western blot. **C** and **D** KGN cells were treated with 5 or 10 ng/mL TGF-β1 for 6 h. The SPARC mRNA levels (**C**) and protein levels (**D**) were examined by RT-qPCR and western blot, respectively. **E** Primary hGL cells were treated with 5 ng/mL TGF-β1 (T) for 6, 12, and 24 h, and the protein levels of SPARC were examined by western blot. The results are expressed as the mean ± SEM of at least three independent experiments. Values that are statistically different from one another (*p* < 0.05) are indicated by different letters

To make our study more technically feasible, especially for those gene knockdown experiments, we used KGN, a cell line derived from human ovarian granulosa cell tumors, as our in vitro model. The KGN cell line has been widely used because it preserves various physiological functions of normal granulosa cells [23]. The human follicular fluid provides an important microenvironment for maintaining the physiological functions of the ovarian follicle. It has been shown that the concentration of TGF-β1 in the human follicular fluid can reach 18.03 ng/mL [27]. Therefore, to examine the effect of TGF-β1 on SPARC expression, KGN cells were treated with 10 ng/mL TGF-β1. As shown in Fig. 1A, treatment of TGF-β1 for 3, 6, 12, and 24 h upregulated SPARC mRNA levels with the maximal effect observed after 6 h of treatment (Fig. 1A). Western blot results showed that 6 h of TGF-β1 treatment upregulated SPARC protein levels, and the stimulatory effect of TGF-β1 remained detectable after 24 h of treatment (Fig. 1B). We also tested the effect of different TGF-β1 concentrations on SPARC expression. As shown in Fig. 1C and D, 5 and 10 ng/mL TGF-β1 induced comparable SPARC mRNA and protein levels in KGN cells. Therefore, 5 ng/mL TGF-β1 was used for ensuing experiments. We next confirmed the stimulatory effect of TGF-β1 on SPARC expression in primary cultures of hGL cells obtained from patients undergoing IVF treatment. As shown in Fig. 1E, treatment with TGF-β1 for 6, 12, or 24 h significantly upregulated SPARC protein levels. The maximal stimulatory effect of TGF-β1 on SPARC protein levels was observed after 24 h of treatment.

### TGF-β1 upregulates SPARC expression through the ALK5-SMAD3 signaling pathway

**Fig. 2:**
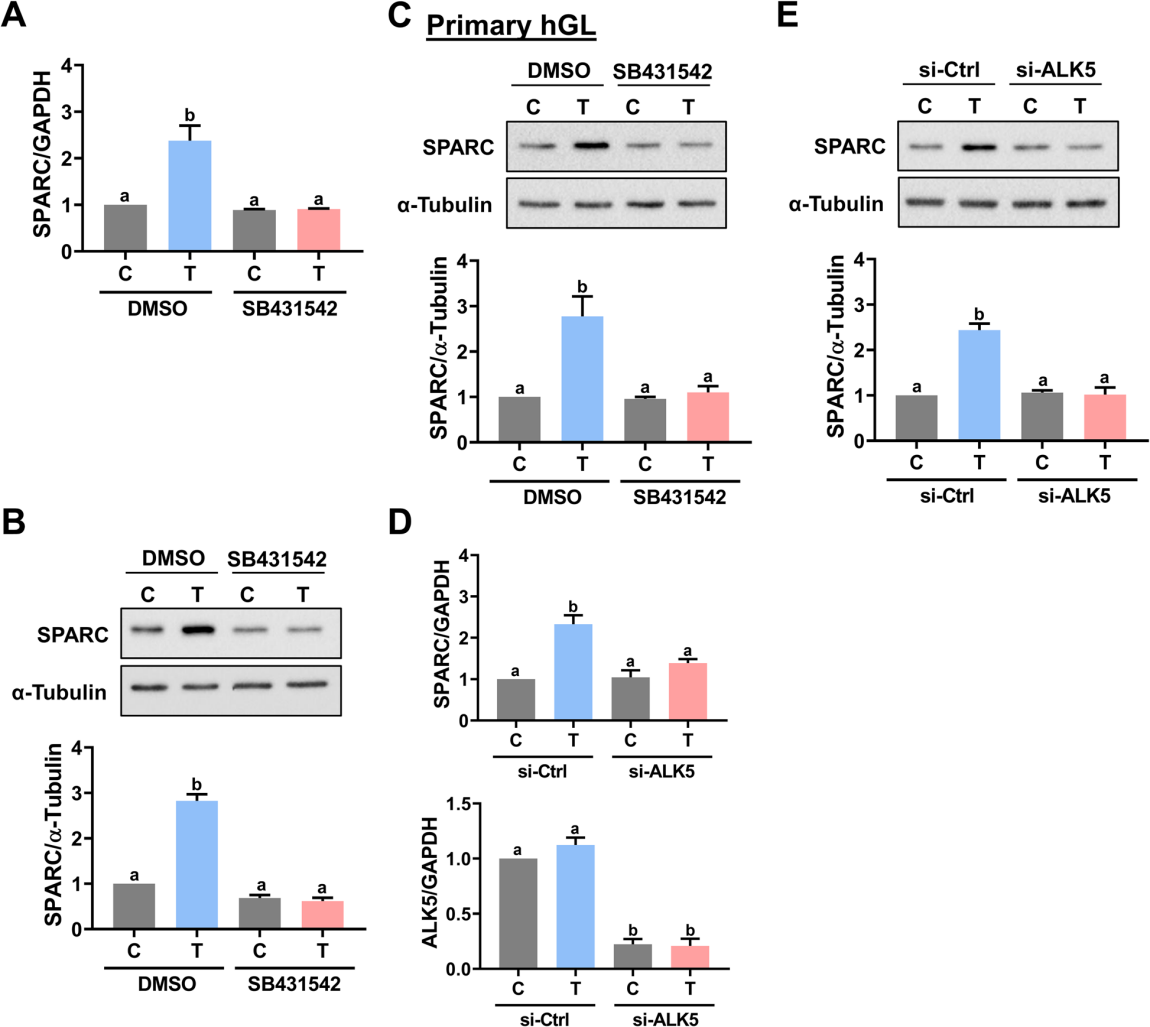
ALK5 mediates TGF-β1-induced SPARC expression. **A** and **B**, KGN cells were pretreated with vehicle control (DMSO) or 10 µM SB431542 for 1 h, and then treated with 5 ng/mL TGF-β1 (T) for 6 h. The SPARC mRNA levels (**A**) and protein levels (**B**) were examined by RT-qPCR and western blot, respectively. **C**, Primary hGL cells were pretreated with vehicle control (DMSO) or 10 µM SB431542 for 1 h, and then treated with 5 ng/mL TGF-β1 (T) for 6 h. The SPARC protein levels were examined by western blot. **D** and **E** KGN cells were transfected with 50 nM control siRNA (si-Ctrl) or ALK5 siRNA (si-ALK5) for 48 h, and then treated with 5 ng/mL TGF-β1 (T) for 6 h. The SPARC mRNA levels (**D**) and protein levels (**E**) were examined by RT-qPCR and western blot, respectively. The results are expressed as the mean ± SEM of at least three independent experiments. Values that are statistically different from one another (*p* < 0.05) are indicated by different letters

**Fig. 3:**
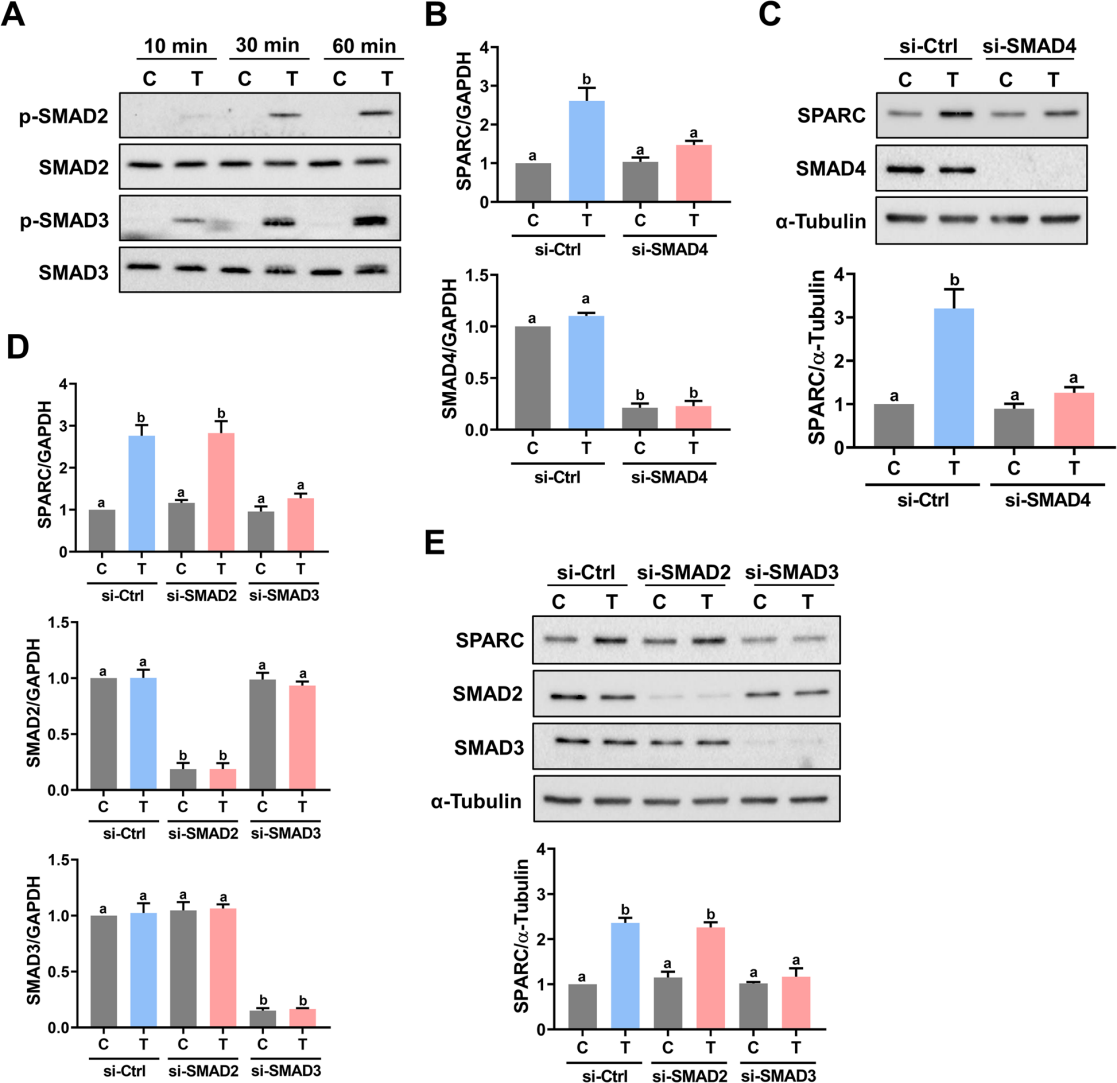
SMAD3 is required for TGF-β1-induced SPARC expression. **A** KGN cells were treated with 5 ng/mL TGF-β1 (T) for 10, 30, and 60 min. The levels of phosphorylated and total forms of SMAD2 and SMAD3 were determined by western blot. **B** and **C** KGN cells were transfected with 50 nM control siRNA (si-Ctrl) or SMAD4 siRNA (si-SMAD4) for 48 h, and then treated with 5 ng/mL TGF-β1 (T) for 6 h. The SPARC mRNA levels (**B**) and protein levels (**C**) were examined by RT-qPCR and western blot, respectively. **D** and **E**, KGN cells were transfected with 50 nM control siRNA (si-Ctrl), SMAD2 siRNA (si-SMAD2) or SMAD3 siRNA (si-SMAD3) for 48 h, and then treated with 5 ng/mL TGF-β1 (T) for 6 h. The SPARC mRNA levels (**D**) and protein levels (**E**) were examined by RT-qPCR and western blot, respectively. The results are expressed as the mean ± SEM of at least three independent experiments. Values that are statistically different from one another (*p* < 0.05) are indicated by different letters

TGF-β1 signals through heteromeric complexes of TGF-β type-I (TβRI) and type-II (TβRII) serine/threonine kinase receptors. When TGF-β1 binds to the TβRII, the TβRI is recruited and phosphorylated, which in turn activates the intracellular SMAD proteins SMAD2 and SMAD3. Activated SMAD2 or SMAD3 forms a heterocomplex with SMAD4, the co-SMAD protein. The SMAD complexes translocate into the nucleus and bind to the SMAD-specific binding element of TGF-β1 target genes to regulate their expressions [28, 29]. TβRI is also known as ALK5. Pretreatment of ALK5 inhibitor SB431542 blocked the stimulatory effect of TGF-β1 on SPARC mRNA levels in KGN cells (Fig. 2A). Western blot analysis showed similar results that inhibition of ALK5 abolished the stimulatory effect of TGF-β1 on SPARC protein levels (Fig. 2B). Consistent with the results obtained from KGN cells, inhibition of ALK5 by SB431542 blocked the stimulatory effect of TGF-β1 on SPARC protein levels in primary hGL cells (Fig. 2C). To avoid any off-target effect of the pharmacological inhibitor, we used a siRNA-mediated knockdown approach to confirm the involvement of ALK5 in TGF-β1-induced SPARC expression. As shown in Fig. 2D, transfection of KGN cells with ALK5 siRNA significantly downregulated endogenous ALK5 mRNA levels. Knockdown of ALK5 abolished the TGF-β1-induced upregulation of SPARC mRNA levels. Similarly, the TGF-β1-stimulated SPARC protein levels were abolished by the knockdown of ALK5 in KGN cells (Fig. 2E). Treatment of TGF-β1 activated SMAD2 and SMAD3 in KGN cells (Fig. 3A). To examine the involvement of SMAD signaling in TGF-β1-induced SPARC expression, the SMAD4 was knocked down by transfecting cells with SMAD4 siRNA. As shown in Fig. 3B and C, the knockdown of SMAD4 attenuated the stimulatory effects of TGF-β1 on SPARC mRNA and protein levels. In a context-dependent manner, SMAD2 and SMAD3 mediate TGF-β1-regulated biological function redundantly or differentially, although they are highly homologous [30]. To examine the requirement of SMAD2 and SMAD3 for the TGF-β1-induced SPARC expression, siRNA was used to knock down SMAD2 or SMAD3 individually. As shown in Fig. 3D, the siRNA-mediated knockdown of SMAD2 specifically downregulated the endogenous SMAD2 mRNA levels without affecting the endogenous SMAD3 mRNA levels and vice versa for SMAD3 siRNA-mediated knockdown. RT-qPCR results showed that the knockdown of SMAD2 did not affect the stimulatory effect of TGF-β1 on SPARC mRNA levels. However, the TGF-β1-induced SPARC mRNA levels were attenuated by the knockdown of SMAD3. Western blot results confirmed that SMAD3, but not SMAD2, was required for the TGF-β1-induced SPARC protein levels in KGN cells (Fig. 3E).

### Slug mediates the TGF-β1-induced SPARC expression

**Fig. 4:**
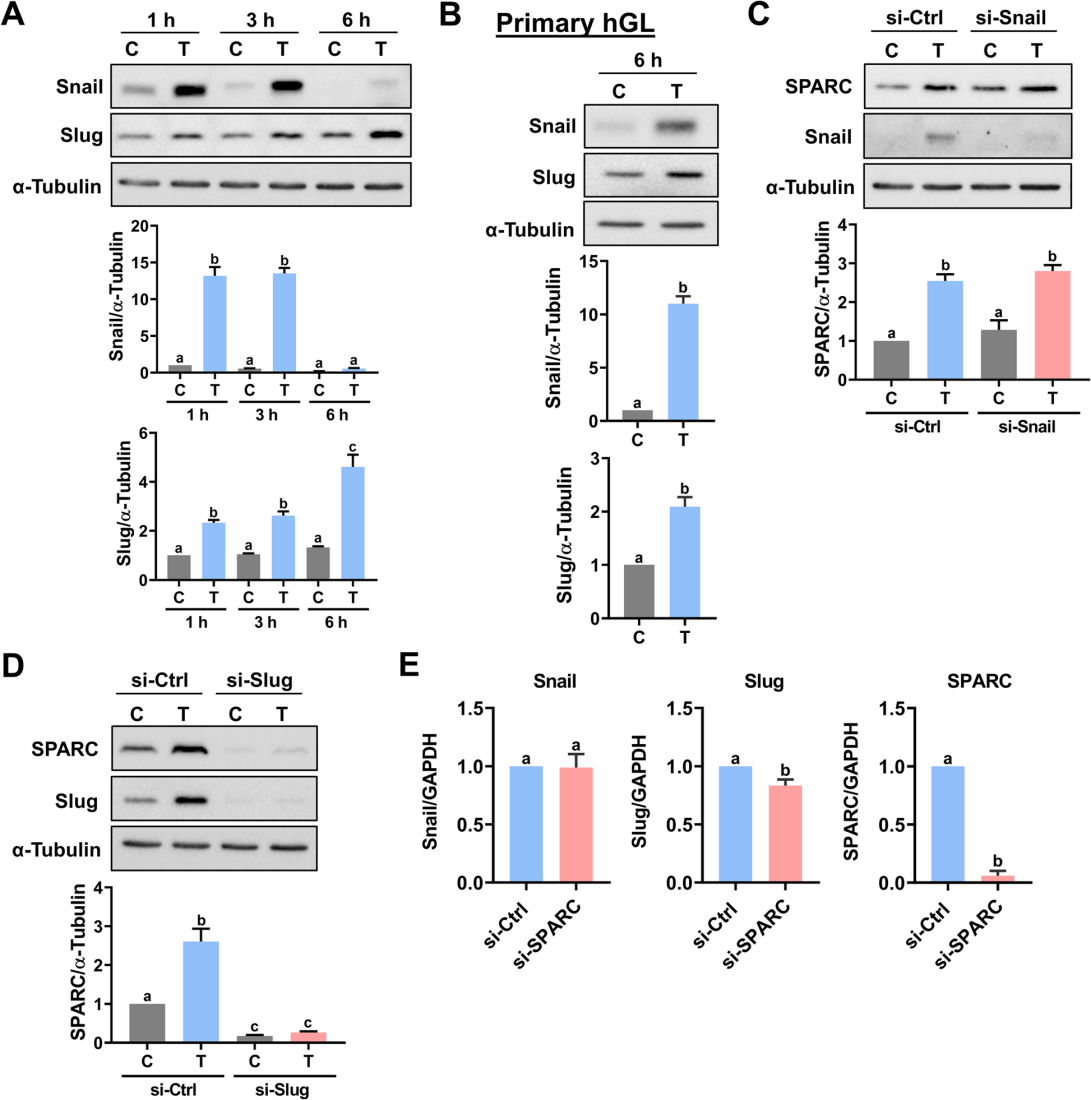
TGF-β1-induced Slug expression is required for the induction of SPARC. **A** KGN cells were treated with 5 ng/mL TGF-β1 (T) for 1, 3, and 6 h, and the protein levels of Snail and Slug were examined by western blot. **B** Primary hGL cells were treated with 5 ng/mL TGF-β1 (T) for 6 h, and the protein levels of Snail and Slug were examined by western blot. **C** and **D** KGN cells were transfected with 50 nM control siRNA (si-Ctrl), Snail siRNA (si-Snail) (**C**) or Slug siRNA (si-Slug) (**D**) for 48 h, and then treated with 5 ng/mL TGF-β1 (T) for 6 h. The SPARC protein levels were examined by western blot. **E** KGN cells were transfected with 50 nM control siRNA (si-Ctrl) or SPARC siRNA (si-SPARC) for 48 h. The Snail and Slug mRNA levels were examined by RT-qPCR. The results are expressed as the mean ± SEM of at least three independent experiments. Values that are statistically different from one another (*p* < 0.05) are indicated by different letters

The Snail and Slug are transcription factors and are well-known to mediate TGF-β1-induced downregulation of E-cadherin expression by binding to the E-box in the E-cadherin promoter [31]. In mice, Snail and Slug are expressed in the ovary and have been suggested to play important roles during folliculogenesis, luteinization, and early embryonic development [32, 33]. The SPARC promoter contains a Snail binding site, and Snail is involved in TGF-β1-stimulated SPARC expression in renal cell carcinoma cells. Whether Snail or Slug is required for the TGF-β1-induced SPARC expression in hGL cells remains unknown [34]. Treatment of KGN cells with TGF-β1 for 1, 3, and 6 h significantly induced Snail and Slug protein levels in a time-dependent manner (Fig. 4A). The stimulatory effects of TGF-β1 on Snail and Slug protein expressions were also observed in the hGL cells (Fig. 4B). We next examine whether Snail or Slug is required for the TGF-β1-induced SPARC expression. KGN cells transfected with Snail siRNA blocked the TGF-β1-induced Snail protein levels. However, the TGF-β1-induced SPARC protein levels were not affected by the knockdown of Snail (Fig. 4C). Interestingly, siRNA-mediated knockdown of Slug downregulated basal and blocked the TGF-β1-induced SPARC protein levels (Fig. 4D). In addition to the stimulatory effect of TGF-β1 on SPARC expression, Snail and Slug can also be upregulated by SPARC in human cancer cells [35–38]. To examine whether the same is true in hGL cells, SPARC was knocked down in the KGN cells, and the mRNA levels of Snail and Slug were examined. As shown in Fig. 4E, transfection of SPARC siRNA significantly downregulated endogenous SPARC mRNA levels. Knockdown of SPARC did not affect the Snail mRNA levels. Although it was statistically significant, the knockdown of SPARC only slightly decreased the mRNA levels of Slug. Collectively, these results indicate that TGF-β1-induced Slug expression is required for the induction of SPARC in hGL cells.

### SPARC and Slug are upregulated in the ovaries of OHSS rats

**Fig. 5:**
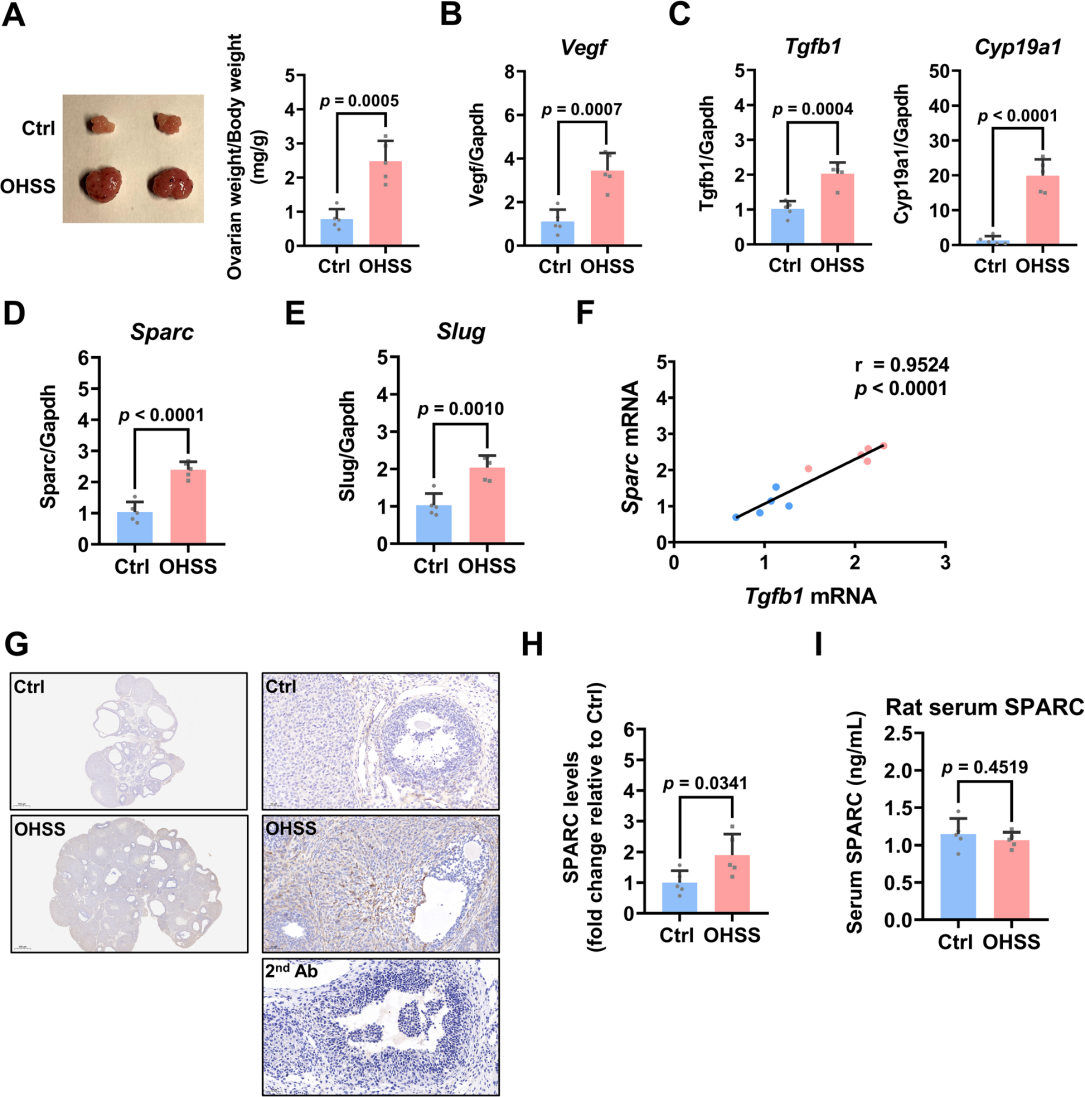
SPARC is upregulated in the ovaries of OHSS rats. **A** Representative rat ovaries were photographed from control (Ctrl) and OHSS groups (left panel). Ovarian weight and body weight were measured in both Ctrl (*n* = 5) and OHSS (*n* = 5) groups after OHSS induction. **B**-**E** The mRNA levels of Vegf (**B**), Tgfb1 and Cyp19a1 (**C**), Sparc (**D**), and Slug (**E**) in ovaries of Ctrl and OHSS rats were examined by RT-qPCR. **F** Pearson’s correlation analysis was performed to evaluate the correlation between Tgfb1 and Sparc mRNA levels in Ctrl and OHSS rats. **G** Representative images of immunostaining for SPARC in rat ovarian sections. Original magnification: 20 × and 200x. The scale bar represents 500 μm and 50 μm. **H** Quantification results for SPARC in Ctrl and OHSS rat ovaries. **I** SPARC protein levels in the rat serum were examined by ELISA. The results are expressed as the mean ± SD

To explore the role of SPARC in OHSS, we examined the expression of SPARC and Slug in the ovaries of OHSS rats. Consistent with our previous studies [19, 26], induction of OHSS enlarged the size of the ovaries and increased ovarian weight in rats (Fig. 5A). As expected, RT-qPCR results showed that Vegf mRNA levels were significantly upregulated in the ovaries of OHSS rats (Fig. 5B). In addition, consistent with our previous results, Tgfb1 and Cyp19a1 mRNA levels were also increased in the OHSS rat ovaries (Fig. 5C). As shown in Fig. 5D and E, the upregulations of Sparc and Slug mRNA levels were detected in the OHSS rat ovaries. Correlation analysis results showed that in control and OHSS rats, the mRNA levels of ovarian Tgfb1 were positively correlated with Sparc mRNA levels in the ovaries (Fig. 5F). Immunohistochemistry confirmed the upregulation of SPARC protein levels in the ovaries of OHSS rats (Fig. 5G-H). Similar to previous studies, histological analysis showed that an increase in corpus luteum number was observed in the OHSS group [39, 40]. Interestingly, ELISA results showed that the serum levels of SPARC protein were not varied between control and OHSS rats (Fig. 5I). These results suggest the local role of SPARC in the regulation of the pathogenesis of OHSS.

### SPARC protein levels are upregulated in the follicular fluid of OHSS patients

**Fig. 6:**
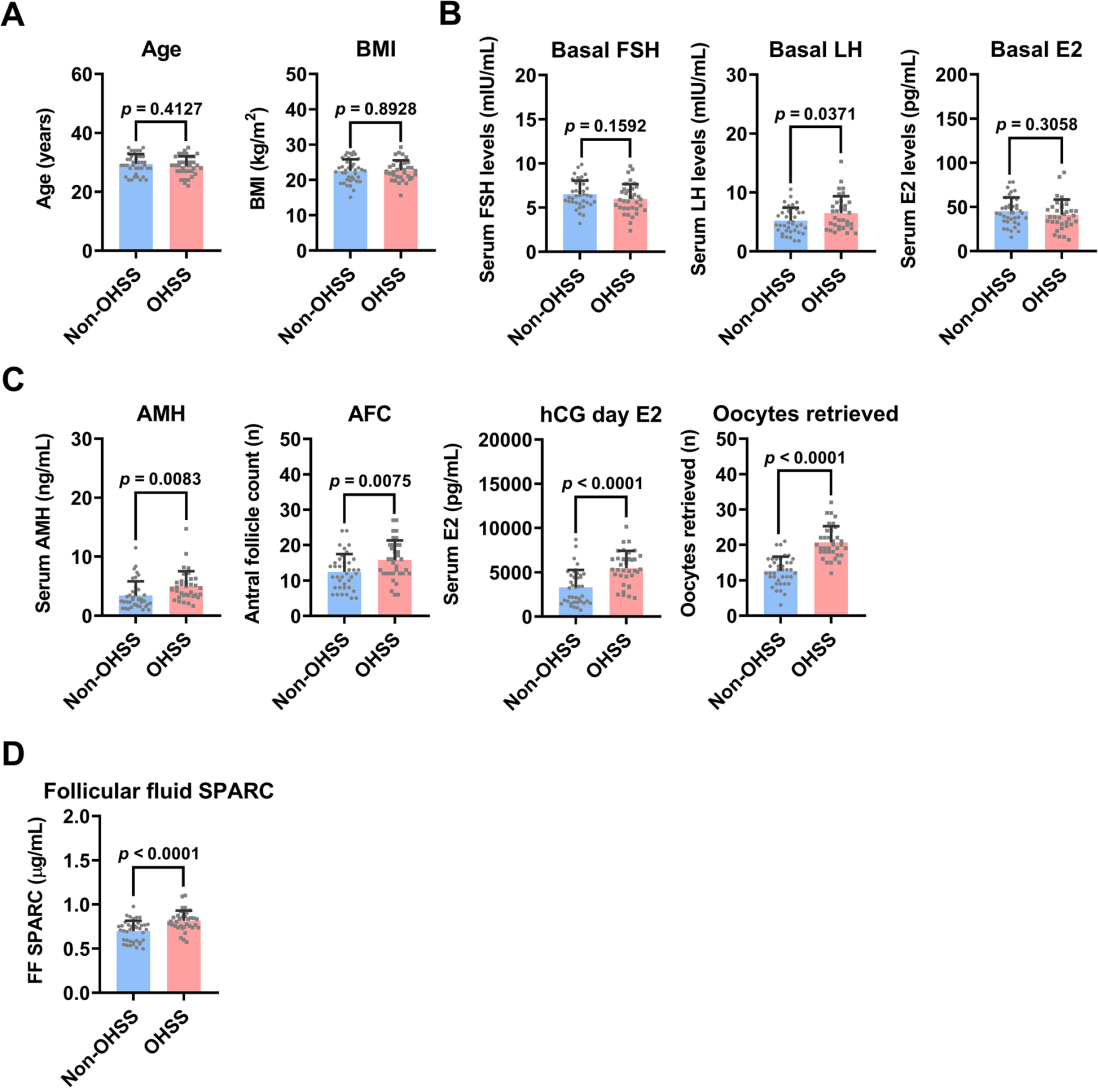
SPARC is upregulated in the follicular fluid of OHSS patients. Follicular fluid samples were collected from 39 non-OHSS and 35 OHSS patients. **A** The age and body mass index (BMI) are presented. **B** The basal serum follicle-stimulating hormone (FSH), luteinizing hormone (LH), and estradiol (E2) levels are presented. **C** The serum levels of anti-müllerian hormone (AMH), antral follicle counts (AFC), serum E2 levels on hCG administration day, and the number of oocytes retrieved are presented. **D** SPARC protein levels in the follicular fluid were examined by ELISA. The results are expressed as the mean ± SD

To examine whether SPARC expression is changed in OHSS patients, 35 follicular fluid samples of OHSS and 39 follicular samples of non-OHSS were collected. In our included patients, no significant differences in age and body mass index (BMI) were observed between non-OHSS and OHSS patients (Fig. 6A). Basal serum FSH and E2 levels were similar between the two groups. However, the basal serum LH levels were higher in OHSS patients than in non-OHSS patients (Fig. 6B). Agree with the well-known characteristics of OHSS, the serum anti-müllerian hormone (AMH) levels, antral follicle counts (AFC), serum E2 levels on hCG administration day, and the number of oocytes retrieved were significantly higher in OHSS patients than in non-OHSS patients (Fig. 6C). ELISA analysis showed that the expression of SPARC protein was detected in human follicular fluid. Importantly, the follicular fluid SPARC levels were significantly upregulated in OHSS patients (0.814 ± 0.116 µg/mL) compared to non-OHSS patients (0.693 ± 0.119 µg/mL) (Fig. 6D).

### SPARC mediates TGF-β1-induced upregulation of VEGF and aromatase

**Fig. 7:**
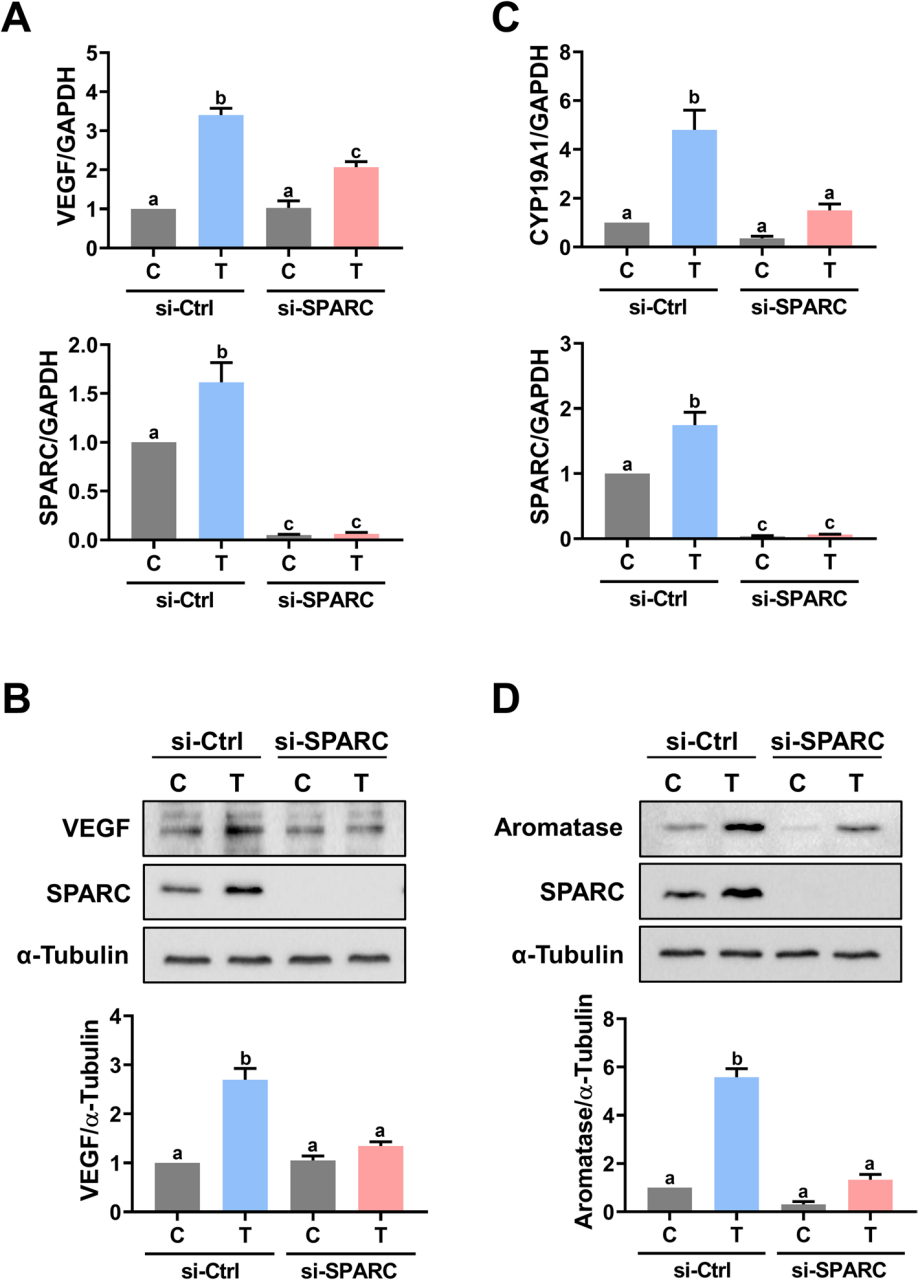
SPARC is required for TGF-β1-stimulated VEGF and aromatase expression in KGN cells. **A** and **B** KGN cells were transfected with 50 nM control siRNA (si-Ctrl) or SPARC siRNA (si-SPARC) for 48 h, and then treated with 5 ng/mL TGF-β1 (T) for 3 h. The VEGF mRNA levels (**A**) and protein levels (**B**) were examined by RT-qPCR and western blot, respectively. **C** and **D** KGN cells were transfected with 50 nM control siRNA (si-Ctrl) or SPARC siRNA (si-SPARC) for 48 h, and then treated with 5 ng/mL TGF-β1 (T) for 24 h. The aromatase (CYP19A1) mRNA levels (**C**) and protein levels (**D**) were examined by RT-qPCR and western blot, respectively. The results are expressed as the mean ± SEM of at least three independent experiments. Values that are statistically different from one another (*p* < 0.05) are indicated by different letters

**Fig. 8:**
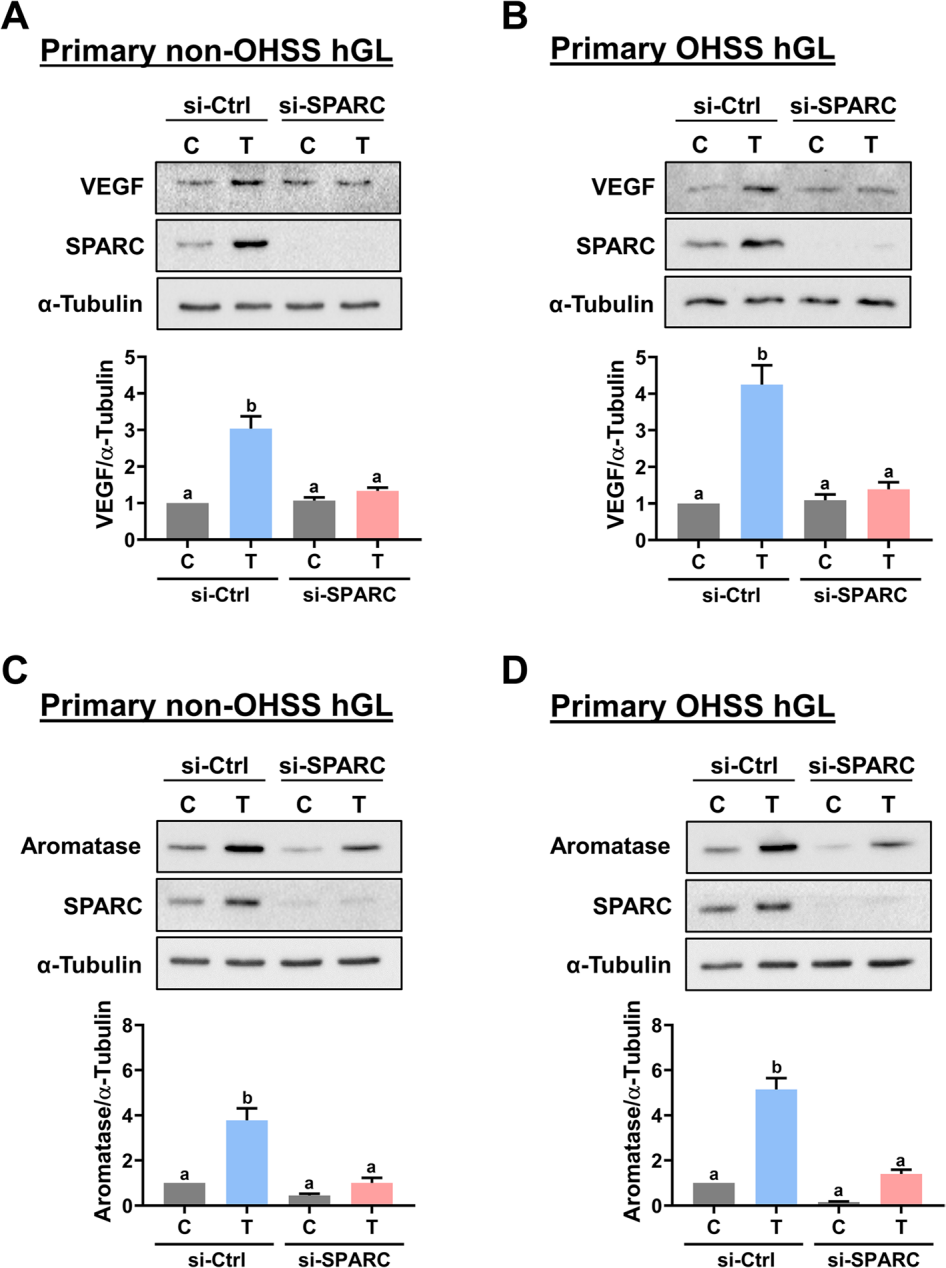
SPARC is required for TGF-β1-stimulated VEGF and aromatase expression in both primary hGL cells derived from control and OHSS patients. **A** and **B** Primary hGL cells derived from non-OHSS (**A**) and OHSS (**B**) were transfected with 50 nM control siRNA (si-Ctrl) or SPARC siRNA (si-SPARC) for 48 h, and then treated with 5 ng/mL TGF-β1 (T) for 3 h. The VEGF protein levels were examined by western blot. **C** and **D** Primary hGL cells derived from non-OHSS (**C**) and OHSS (**D**) were transfected with 50 nM control siRNA (si-Ctrl) or SPARC siRNA (si-SPARC) for 48 h, and then treated with 5 ng/mL TGF-β1 (T) for 24 h. The aromatase protein levels were examined by western blot. The results are expressed as the mean ± SEM of at least three independent experiments. Values that are statistically different from one another (*p* < 0.05) are indicated by different letters

Given the stimulatory effects of TGF-β1 on VEGF and aromatase expressions in hGL cells and their roles in the pathogenesis of OHSS, we next examined the involvements of SPARC in TGF-β1-induced upregulation of VEGF and aromatase. As shown in Fig. 7A and B, treatment of KGN cells with TGF-β1 increased VEGF mRNA and protein levels. The TGF-β1-induced upregulation of VEGF mRNA and protein levels were attenuated by the siRNA-mediated knockdown of SPARC. Similar to VEGF, the expression levels of aromatase mRNA and protein were upregulated in KGN cells in response to the TGF-β1 treatment. Knockdown of SPARC attenuated the TGF-β1-stimulated aromatase mRNA and protein levels (Fig. 7C and D). To further confirm the involvement of SPARC in TGF-β1-induced VEGF and aromatase expressions, primary hGL cells derived from both non-OHSS and OHSS patients were used. As shown in Fig. 8A and B, treatment of TGF-β1 upregulated protein levels of VEGF in primary hGL cells derived from both non-OHSS and OHSS patients. In addition, the stimulatory effect of TGF-β1 on VEGF protein levels were blocked by the siRNA-mediated knockdown of SPARC. Similarly, the knockdown of SPARC attenuated the TGF-β1-induced aromatase protein levels in primary hGL cells derived from both non-OHSS and OHSS patients (Fig. 8C and D). Taken together, these results suggest that the increase of SPARC expression could contribute to OHSS development by mediating the expression of VEGF and aromatase.

### Knockdown of SPARC decreases SMAD4 expression

**Fig. 9:**
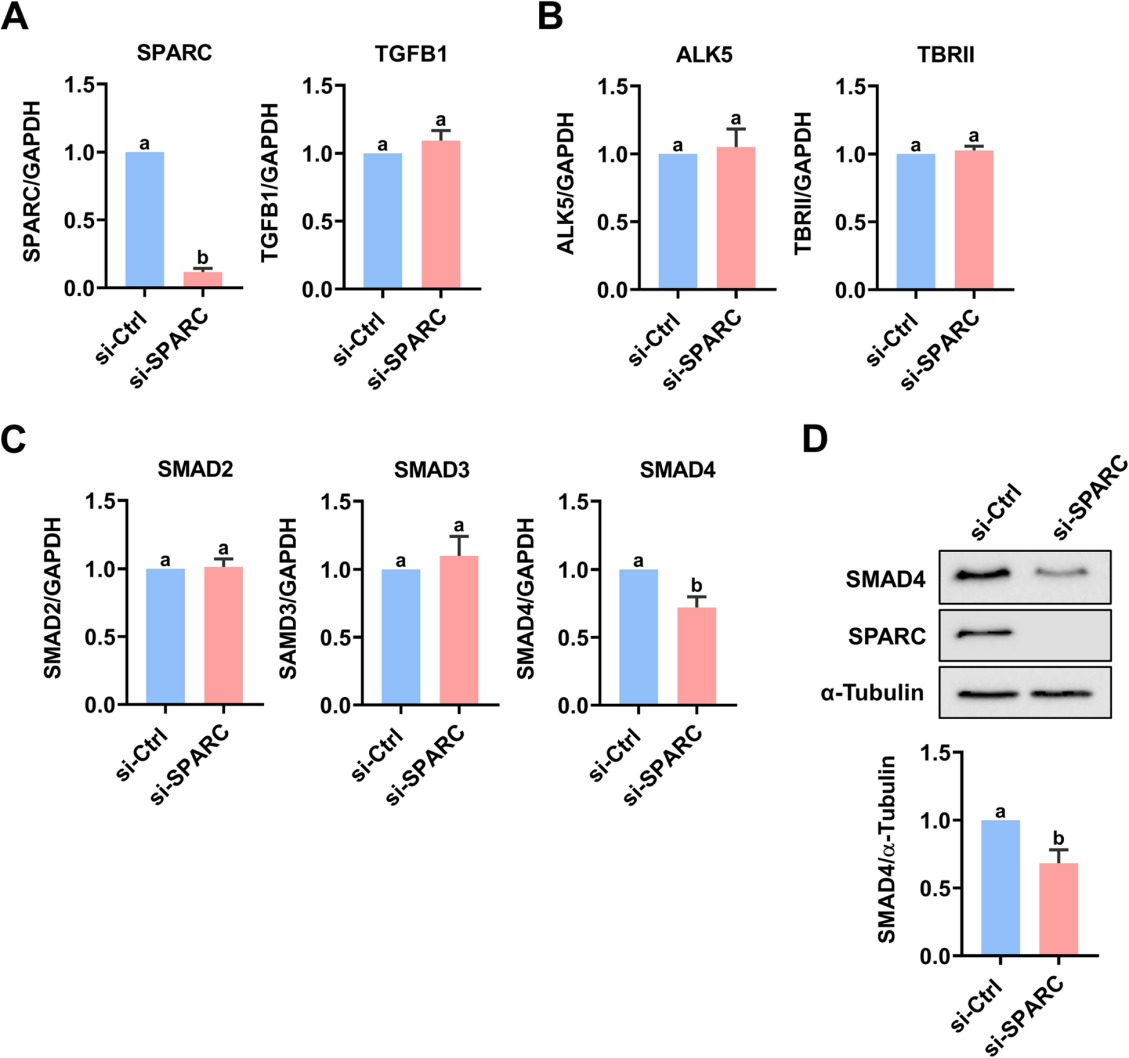
SPARC knockdown downregulates SMAD4 expression. **A**-**C** KGN cells were transfected with 50 nM control siRNA (si-Ctrl) or SPARC siRNA (si-SPARC) for 48 h. The mRNA levels of SPARC and TGFB1 (**A**), ALK5 and TβRII (**B**), SMAD2, SMAD3, and SMAD4 (**C**) were examined by RT-qPCR. **D** KGN cells were transfected with 50 nM control siRNA (si-Ctrl) or SPARC siRNA (si-SPARC) for 48 h. The protein levels of SMAD4 were examined by western blot. The results are expressed as the mean ± SEM of at least three independent experiments. Values that are statistically different from one another (*p* < 0.05) are indicated by different letters

SPARC has been shown to regulate the expression of TGF-β1 in mesangial cells [41, 42]. To examine whether the SPARC knockdown affects the TGF-β1 signaling, KGN cells were transfected with SPARC siRNA, and the expressions of molecules related to TGF-β1 signaling were examined. As shown in Fig. 9A, the knockdown of SPARC did not affect the TGFB1 mRNA levels. In addition, the mRNA levels of TGF-β1 type I and type II receptors, ALK5 and TβRII, were also not affected by the SPARC knockdown (Fig. 9B). We next examined whether expressions of SMAD proteins are changed in response to the SPARC knockdown. As shown in Fig. 9C, the knockdown of SPARC did not affect the SMAD2 and SMAD3 mRNA levels but decreased the mRNA levels of SMAD4 in KGN cells. The inhibitory effect of SPARC knockdown on SMAD4 protein levels was confirmed by the western blot analysis (Fig. 9D). Collectively, these results suggest that the knockdown of SPARC attenuated TGF-β1 signaling by decreasing the SMAD4 expression.

## Discussion

The expression of SPARC in the ovary has been described in different species. In humans, previous studies demonstrate several tumor-suppressive roles of SPARC in ovarian cancer cells [43]. There are two studies that report the expression of SPARC in human ovaries. The mRNA levels of SPARC are reduced in post-menopausal ovaries [44]. The expression of SPARC is downregulated in granulosa cells obtained from PCOS patients with insulin resistance when compared to normal controls [45]. However, the biological function of ovarian SPARC in the non-tumor context remains undetermined. SPARC is a secreted matricellular glycoprotein. In the present study, our ELISA results, for the first time, reported the presence of SPARC protein in the human follicular fluid. In addition, we showed that the expression of SPARC was upregulated by the TGF-β1 treatment in hGL cells. Moreover, the siRNA-mediated knockdown of SPARC attenuated the TGF-β1-stimulated VEGF and aromatase expression. These results suggest that the upregulation of SPARC in the ovaries could contribute to the pathogenesis of OHSS by increasing the expression of VEGF and E2 levels. Therefore, a future study by manipulating the expression of ovarian SPARC in the rat OHSS model can be conducted to investigate the role of SPARC in the development and progression of OHSS.

TGF-β1 can stimulate SPARC expression in different types of cells [46–52]. We showed that SPARC expression was upregulated in hGL cells by TGF-β1. Mechanistically, using the siRNA-mediated knockdown approach, our results demonstrated that SMAD3 but not SMAD2 was involved in the TGF-β1-induced SPARC expression in hGL cells. Our previous studies showed that induction of VEGF, connective tissue growth factor, and cyclooxygenase-2 by TGF-β1 in hGL cells requires both SMAD2 and SMAD3 [18, 53, 54]. TGF-β1 downregulates steroidogenic acute regulatory protein expression in hGL cells through SMAD3 but not SMAD2 [55]. SMAD2 but not SMAD3, mediates the TGF-β1-induced aromatase expression in hGL cells [20]. Collectively, these results indicate that the requirement of SMAD2 or SMAD3 for the TGF-β1 function is in a target gene-dependent manner in hGL cells. In addition to the canonical SMAD2/3 signaling, TGF-β1 activates non-canonical signaling pathways such as ERK1/2 and PI3K/AKT [56]. Whether these signaling pathways are involved in the TGF-β1-induced SPARC expression remains undetermined and warrants further investigations. Interestingly, SPARC can also regulate the expression of TGF-β1 in mesangial cells [41, 42]. Therefore, we examined whether the same effect could be observed in hGL cells. Our results showed that the knockdown of SPARC did not affect the expression of TGF-β1 in hGL cells. This observation indicates that in hGL cells SPARC expression is upregulated by TGF-β1, but TGF-β1 expression is not affected by SPARC.

Induction of Snail and Slug transcription factors plays a key role in TGF-β1-induced epithelial-mesenchymal transition. The binding site for Snail and Slug has been identified in the SPARC promoter, and Snail is required for the TGF-β1-induced SPARC expression in renal cell carcinoma cells [34]. Knockdown of Snail decreases SPARC expression in human breast carcinoma MDA-MB-231 cells [57]. In the present study, both Snail and Slug expressions were upregulated by TGF-β1 in hGL cells. However, in contradiction to previous studies, we found that TGF-β1-induced SPARC expression required Slug but not Snail expression in hGL cells. These conflicting results may be attributed to the different types of cells. Depending on the cancer type, the expression of Snail, Slug, or both can be upregulated by SPARC [35–38]. Using a siRNA-mediated knockdown approach, we showed that the knockdown of SPARC did not affect the Snail expression but downregulated the expression of Slug. These results together with the stimulatory effect of Slug on SPARC expression, suggest the existence of a positive feedback loop between Slug and SPARC in hGL cells. The binding sites for other transcription factors, such as AP-1 and SP-1, are identified in the SPARC promoter [58]. It is still unclear whether AP-1 or SP-1 participates in TGF-β1-induced SPARC expression in hGL cells. Thus, the investigation of transcriptional regulation of SPARC induced by TGF-β1 will be of great interest.

The levels of TGF-β1 in follicular fluid are upregulated in OHSS patients, and that contributes to the development of OHSS [18]. In this study, for the first time, we found that SPARC expression was upregulated in rat OHSS ovaries and follicular fluid of OHSS patients. Our results also revealed that SPARC was involved in the TGF-β1-induced expression of VEGF and aromatase. Angiogenesis and vascular hyperpermeability have been reported to be the key factors for inducing OHSS. VEGF acts as an angiogenic and vasoactive factor that increase angiogenesis and vascular permeability. Therefore, VEGF is considered the most important factor that mediates the pathogenesis of OHSS. We showed that the knockdown of SPARC attenuated the TGF-β1-stimulated VEGF expression in hGL cells, which suggests the involvement of SPARC in the pathogenesis of OHSS. Although SPARC is required for the TGF-β1-induced VEGF expression in hGL cells, the precise mechanism behind this regulation is unclear. Our results showed that the knockdown of SPARC decreased the SMAD4 expression, which could result in a reduction of TGF-β1 signaling. This finding could explain the results that the knockdown of SPARC attenuated TGF-β1-induced VEGF and aromatase expression in hGL cells. In addition to expression, SPARC can block VEGFR-1-mediated recruitment of the phosphatase SHP-1 to VEGFR-2, thereby allowing VEGF-induced phosphorylated VEGFR-2 to activate an angiogenic response [59]. Therefore, it is possible that increases in SPARC expression enhance VEGF/VEGFR-2 signaling in hGL and contribute to the development of OHSS.

Several clinical indexes have been used to predict the development of OHSS [60, 61]. However, there is no single method to prevent OHSS completely. Therefore, discovering a novel biomarker would help to early prevent the development of OHSS. Human follicular fluid provides an important microenvironment for maintaining the physiological functions of the ovarian follicle. We have shown that the expression levels of an EGFR ligand, amphiregulin, and TGF-β1 are upregulated in the follicular fluid of OHSS patients when compared to control patients [18, 62, 63]. In the present study, we identified the expression of SPARC protein in the follicular fluid and found that OHSS patients had higher follicular fluid SPARC levels than non-OHSS patients. As the follicular fluid is collected from patients for oocyte retrieval, it is feasible to use follicular SPARC as a biomarker together with other known indexes of biomarkers to predict patients who will subsequently develop OHSS.

## Conclusions

In summary, the present study demonstrates that SPARC expression is upregulated by TGF-β1 in hGL cells. The stimulatory effect of TGF-β1 on SPARC expression is mediated by SMAD3 signaling. Our results also reveal that TGF-β1 treatment induces expressions of transcription factors, Snail and Slug. However, only Slug is involved in TGF-β1-induced SPARC expression. In addition, the knockdown of SPARC decreases Slug expression suggesting a positive feedback loop exists between Slug and SPARC. Moreover, SPARC expression is upregulated in the ovaries of OHSS rats and the follicular fluid of OHSS patients. Knockdown of SPARC attenuates TGF-β1-induced VEGF and aromatase expression by decreasing SMAD4 expression, indicating the involvement of SPARC in the development of OHSS. This study provides a novel mechanism for the regulation of SPARC in hGL cells and increases the understanding of the physiological and pathological roles of SPARC in the ovary, which could help to develop therapeutic methods for the OHSS.

## Supplementary Information

**Additional file 1.**

## Data Availability

The data that support the findings of this study are available from the corresponding author upon reasonable request.

## Abbreviations

AMH: Anti-müllerian hormone
AFC: Antral follicle counts
BMI: Body mass index
hCG: Human chorionic gonadotropin
E2: Estradiol
FSH: Follicle-stimulating hormone
hGL: Human granulosa-lutein cells
IVF: In vitro fertilization
LH: Luteinizing hormone
PMSG: Pregnant mare serum gonadotropin
OHSS: Ovarian hyperstimulation syndrome
RT-qPCR: Reverse transcription quantitative real-time PCR
SPARC: Secreted protein acidic and rich in cysteine
siRNA: Small interfering RNA
TβRI: TGF-β type-I receptor
TβRII: TGF-β type-II receptor
TGF-β1: Transforming growth factor-beta 1
TBS: Tris-buffered saline
VEGF: Vascular endothelial growth factor
