# Supplementary material for: TGF-β1 upregulates secreted protein acidic and rich in cysteine expression in human granulosa-lutein cells: a potential mechanism for the pathogenesis of ovarian hyperstimulation syndrome
Source: Cell Commun Signal. 2023 May 8;21:101. doi: 10.1186/s12964-023-01123-2 (PMC10165787; doi:10.1186/s12964-023-01123-2)
Supplement: Supplementary file 1 [file 12964_2023_1123_MOESM1_ESM.docx]

Gene	Forward	Reverse
**Human**		
ALK5	GTTAAGGCCAAATATCCCAAACA	ATAATTTTAGCCATTACTCTCAAGG
CYP19A1	GAGAATTCATGCGAGTCTGGA	CATTATGTGGAACATACTTGAGGACT
GAPDH	GAGTCAACGGATTTGGTCGT	GACAAGCTTCCCGTTCTCAG
Slug	TTCGGACCCACACATTACCT	GCAGTGAGGGCAAGAAAAAG
SMAD2	CCGAAATGCCACGGTAGAAA	GGGCTCTGCACAAAGATTGC
SMAD3	CCCCAGCACATAATAACTTGG	AGGAGATGGAGCACCAGAAG
SMAD4	TCCACAGGACAGAAGCCATT	GTCACTAAGGCACCTGACCC
Snail	CCCCAATCGGAAGCCTAACT	GCTGGAAGGTAAACTCTGGATTAGA
SPARC	GCTCCACCTGGACTACATCG	TGACCAGGACGTTCTTGAGC
TBRII	GCTCTGGTGCTCTGGGAAAT	GAATTTCTGGTCGCCCTCGA
TGFB1	TACCTGAACCCGTGTTGCTCTC	GTTGCTGAGGTATCGCCAGGAA
VEGF	CCCACTGAGGAGTCCAACAT	TGCATTCACATTTGTTGTGC
**Rat**		
Cyp19a1	GCTGGACACTTCTAACACGC	ATAAGGAGTGCTTGCCAGGC
Gapdh	GACATGCCGCCTGGAGAAAC	AGCCCAGGATGCCCTTTAGT
Slug	TGGCTGCTTCAAGGACACAT	GCAGATGTGCCCTCAGGTTT
Sparc	GGCAACAACCTCCTCACTGA	TTCTCAAAGTCTCGGGCCAG
Tgfb1	TCAACTGTGGAGCAACACGT	CACTCAGGCGTATCAGTGGG
Vegf	ACGAAAGCGCAAGAAATCCC	TAACTCAAGCTGCCTCGCC
